# Characterization of HIV-1 recombinant and subtype B near full-length genome among men who have sex with men in South Korea

**DOI:** 10.1038/s41598-021-82872-3

**Published:** 2021-02-18

**Authors:** Sangmi Ryou, Myeongsu Yoo, Kisoon Kim, Sangsoo Kim, Sang Il Kim, Youn Jeong Kim, Dae Won Park, Jun Yong Choi, Hyo Youl Kim, Jung Ho Kim, Joon Young Song, Shin-Woo Kim, Hyun-Ha Chang, Bo Youl Choi, Mee-Kyung Kee

**Affiliations:** 1 Division of Viral Disease Research, Center for Infectious Diseases Research, Korea National Institute of Health, Cheongju, Korea; 2 Department of Bioinformatics and Life Science, Soongsil University, Seoul, Korea; 3 Division of Infectious Disease, Department of Internal Medicine, Seoul St. Mary’s Hospital, College of Medicine, The Catholic University of Korea, Seoul, Korea; 4 Department of Internal Medicine and AIDS Research Institute, Yonsei University College of Medicine, Seoul, Korea; 5 Department of Internal Medicine, Yonsei University Wonju College of Medicine, Wonju, Korea; 6 Division of Infectious Disease, Department of Internal Medicine, Korea University College of Medicine, Seoul, Korea; 7 Department of Internal Medicine, School of Medicine, Kyungpook National University, Daegu, Korea; 8 Department of Preventive Medicine, College of Medicine, Hanyang University, Seoul, Korea

**Keywords:** Genotype, Infectious diseases

## Abstract

In Korea, subtype B is the predominant variant of HIV-1, but full genome sequencing and analysis of its viral variants are lacking. We performed near full-length genome (NFLG) sequencing and phylogenetic and recombination analyses of fifty plasma samples from HIV-positive men who have sex with men (MSM) from a Korea HIV/AIDS cohort study. Viral genomes were amplified and the near-full-length sequences were determined using next-generation sequencing (NGS) and Sanger sequencing. We focused on the HIV-1 subtype classification and identification of HIV recombinants. Twelve HIV-1 NFLGs were determined: ten were subtyped as pure HIV-1 subtype B and two recombinant strains as a common subtype CRF07_BC, and a novel subtype CRF43_02G recombined with CRF02_AG again, or a new CRF02_AG and subtype G recombinant. For the ten NFLGs determined by NGS, “the novel recombinant emerged at approximately 2003 and the other nine subtype B about 2004 or 2005”. This is the first report analyzing HIV-1 NFLG, including recombinants and clinical characteristics, by subtype among MSM in Korea. Our results provide novel insights for understanding the recombinants in the HIV-1 epidemic in Korea.

## Introduction

The human immunodeficiency virus (HIV) is characterized by extremely high genetic variability and rapid evolution. This genetic variability results from the high mutation and recombination rate of reverse transcriptase, which lacks DNA proofreading capacity, together with high rates of viral replication. Insertions and deletions are also common in the HIV genome. This has resulted in high rates of intra- and inter-genetic recombination; therefore, HIV shows a more genetically diverse population with infected hosts^1,2^. Recombinant viruses may contain distinct regions from two or more parental strains owing to simultaneous infection (co-infection) or sequential infection (super infection)^3,4^. HIV-1 recombination impacts many aspects of the HIV pandemic, including viral diversity and fitness, drug resistance, immunological escape, and disease progression. Recombinant viruses already contribute substantially to the global pandemic; currently, 104 circulating recombinant forms (CRFs) have been reported in the Los Alamos National Laboratory HIV Sequence Database (https://www.hiv.lanl.gov/content/sequence/HIV/CRFs/CRFs.html, last update; March 20, 2020). This number will increase as different HIV-1 subtypes are discovered^5^.

The cumulative number of HIV cases in Korea since the first detected person with HIV in 1985 was reportedly 18,725 as of 2019 and the sex ratio was 9.9:1 (male:female). Although cases of HIV infection in Korea are relatively low compared to those in other countries, the number of newly diagnosed cases has increased annually. In addition, the proportion of HIV-diagnosed foreigners in Korea was approximately 11.0% of the total HIV cases. Further, 99.8% of all individuals diagnosed with HIV infection in Korea were infected through sexual contact, and transmission through blood transfusion, vertical transmission, and needle sharing for IDU was infrequent ^6^. For the past 30 years since 1985, the HIV-1 subtype B has been predominant, and the subtypes CRF_01AE, G, and C were observed in previous molecular surveys in Korea. A unique strain of HIV-1 subtype B, known as Korean clade B (Korean B), accounts for > 88% of subtype B infection cases in Korea. Most HIV-1 strains are classified using phylogenetic analysis of small portion genome sequences, such as the *gag, pol,* or *env* genes^7,8^. However, the recombination and sequence diversity of a complex genome cannot be completely characterized when partial genome sequences are used for HIV-1 subtyping. Only full-length sequencing can determine the exact mosaic pattern within a recombinant virus isolate^9^. The aim of the present study was to amplify the full-length HIV-1 genome sequences of clinical isolates in Korea, and to perform genomic characterization through multiple recombination detection methods. To reconstruct an epidemiological history of HIV-1 in Korea, we further performed a Bayesian analysis of these Korean near full genome sequences.

## Results

### Characterization of study samples

Twelve near full-length genomes (NFLGs) were obtained from samples of fifty HIV-positive men who have sex with men (MSM). For twelve patients with NFLG, clinical and epidemiological characteristics are shown in Table 1. Their median age at diagnosis was 32 years (range 21–51); the median CD4^+^ T cell count was 85 cells/mm^3^ (range 7–677); and the median viral load was 210,527 copies/mL (range 67,000–10,000,000); 91.7% (n = 11) received ART. A total of nine patients (75%), who had multiple symptoms, were diagnosed with AIDS-related/-defining disease: HIV-related tuberculosis (TB) (n = 4; 33.3%), HIV-related syphilis (n = 5; 41.7%), oropharyngeal candidiasis (n = 3; 25.0%), and others (gonorrhea n = 1; 8.3%). Furthermore, no differences were observed in the patients of whom full-length genome sequence was not obtained. Supplementary Table S1 shows the epidemiological characteristics of the study population. For the fifty study participants, the median viral load was 145,031 copies/mL (range 63,890–10,000,000); the median CD4^+^ T cell counts was 148 cells/mm^3^ (range 5–677); and forty-two participants (84.0%) received ART at the time of diagnosis.

**Table 1:** Clinical characteristics of twelve HIV-positive men who have sex with men from which near full-length genomes were obtained in Korea.

ID	Age	Diagnosis date	Sampling date	Viral load^a^	CD4+T^a^	CD8+T^a^	ART^a^	Initial ART regimen	AIDS-related/defining diseases^b^
KR001	29	20,060,503	20,070,309	67,000	242	992	Naïve		–
KR002	36	19,981,105	20,070,119	120,000	75	632	Experience		Tuberculosis
KR004	41	20,070,221	20,070,220	220,000	50	255	Naïve		Syphilis
KR005	17	20,070,130	20,070,201	110,000	493	1079	Naïve		Tuberculosis
KR006	41	20,060,630	20,070,306	115,000	268	531	Naïve		Syphilis
KR012	37	20,080,108	20,080,114	306,000	28	–	Naïve		oropharyngeal Candidiasis
KR014	18	20,071,213	20,080,123	550,000	77	931	Naïve	NNRTI	–
KR016	35	20,080,320	20,080,411	160,000	7	195	Naïve		Tuberculosis, Syphilis, Gonorrhea
KR017	29	20,080,429	20,080,427	358,463	92	1500	Naïve		–
KR020	51	20,080,623	20,080,627	201,054	73	1443	Naïve		Tuberculosis, oropharyngeal Candidiasis
KR021	28	20,081,008	20,080,908	10,000,000	677	1209	Naïve		Syphilis, oropharyngeal Candidiasis
KR050	21	20,160,113	20,160,719	254,000	138	1036	Naïve		Syphilis

*ART* antiretroviral therapy, *NNRTI* non-nucleoside reverse transcriptase inhibitor.

^a^Viral load was measured in copies/mL. CD4+T and CD8+T were measured in cells/mm^3^.

^b^Diseases diagnosed at entry of the study.

### Amplification of NFLG

For the NFLG of twelve samples, PCR amplicons of both fragments 1 (5.5 kb) and 2 (3.7 kb) were obtained. As shown in Table 2, using next-generation sequencing (NGS) and Sanger sequencing, the assembly of the overlapping sequence contigs resulted in several different sequences, which were compared to HXB2, and ten sequences by NGS were used to characterize the near full-length HIV-1 genomes (8628–8801 bp), ranging from the 5′-*gag* region to the 3′-*nef* region. Open reading frames were identified for the *gag*, *pol*, and *env* structural genes, and for the *vif*, *vpr*, *vpu*, *nef*, *tat*, and *rev* regulatory and accessory genes. Additionally, Sanger sequencing identified the NFLGs of two samples, KR021 and KR050, that were 9237 bp in length and spanned from the *gag* to the *nef* genes. Almost all sequences (n = 12) were missing at the 5′ long terminal repeat (LTR) and 3′ LTR regions. We also amplified three partial genomes (*gag*, *pol*, and *env*) of samples using nested PCR (Supplementary information).

**Table 2:** Summary of near full-length genomes from 12 HIV-1 samples with next-generation sequencing (NGS) and Sanger sequencing.

ID	Sequencing method	Summary of sequencing results				Sequence position		Analyses		
		Total no. of paired-end reads	Assembled genome size (bp)	Mean coverage depth (x)	HXB2 coverage (%)	Begin	End	jpHMM	REGA	RDP4
KR001	NGS	5777	8756	2328.89	98.6	790	9411	B	B	–
KR002		6003	8728	1349.06	90.0	790	9411	B	B	–
KR004		15,288	8768	2632.06	70.3	790	9411	B	B	–
KR005		11,920	8628	1699.36	95.7	841	9411	A1, G	CRF43_02G, A1, G	CRF02_G, CRF43_02G
KR006		13,515	8785	5196.68	89.0	790	9411	B	B	–
KR012		10,069	8801	2305.98	90.3	790	9407	B	B	–
KR014		5200	8788	1531.72	33.9	790	9408	B	B	–
KR016		5457	8778	1632.23	90.0	790	9411	B	B	–
KR017		3721	8754	499.44	64.0	790	9411	B	B	–
KR020		8930	8768	2328.67	80.5	790	9411	B	B	–
KR021	Sanger	–	9237	–	95.0	658	9511	B	B	–
KR050		–	9237	–	95.0	538	9511	CRF07_BC	CRF07_BC	CRF07_BC

^a^CRF, Circulating recombinant forms; Recombination events detected by jpHMM, REGA and RDP4; REGA, HIV subtyping tool; jpHMM, jumping profile HMM; RDP4, recombination detection program v.4, include RDP, Bootscan, Maxchi, Chimera, SiSscan, 3Seq.

### Recombinant analyses

NFLGs were mostly classified as HIV-1 subtype B (83%; 10/12), followed by recombinant forms (17%; 2/12). For the two recombinants, KR005 was classified as subtype A1 and subtype G recombinant, and KR050 was classified as CRF07_BC using jpHMM. Furthermore, recombination analysis using the REGA program showed that KR005 was a recombinant of CRF43_02G, subtype A1, and subtype G, and the other recombinant, KR050, was identified as CRF07_BC. The remaining ten samples were identified as subtype B. In particular, we also obtained recombinant events and breakpoints (BPs) from KR005 and KR050 that were supported by at least three of the seven selected tests implemented in RDP4. The results of RDP4 showed that KR005 was related to CRF02_G (AB485636) and CRF43_02G (EU697909). KR050 by RDP4 was shown to be related to CRF07_BC. A similar pattern and BP were observed in the sequences using jpHMM, REGA, and RDP4 (Table 2). We also analyzed three partial genomes of PCR-positive samples. The remaining partial genome sequences included the *gag* for 39 samples (78%), *pol* for 32 samples (64%), and *env* for 29 samples (58%). Overall, subtype B was the most prevalent and was classified as Korean subtype B (Korean B) (Supplementary Table S2).

### Maximum likelihood (ML) phylogenetic analyses for NGS sequences

**Figure 1:**
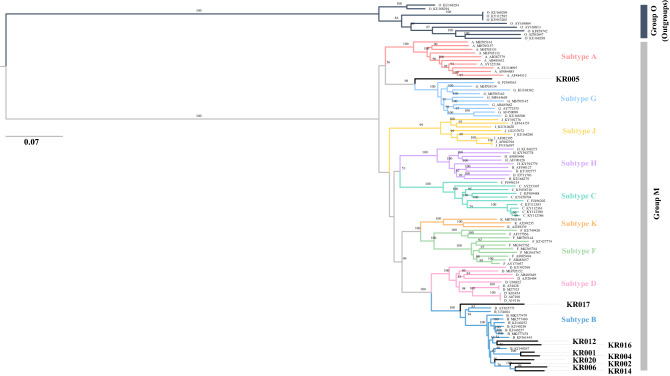
Recombinant analysis of near full-length genome sequences in MSM using phylogenetic analysis. Maximum likelihood tree of NFLG including study sequences in block line. Each subtype reference sequence is indicated in a different color. Numbers associated with tree branches indicate degrees of bootstrap support for these branches and the scale bar represents the number of nucleotide substitutions per site.

**Figure 2:**
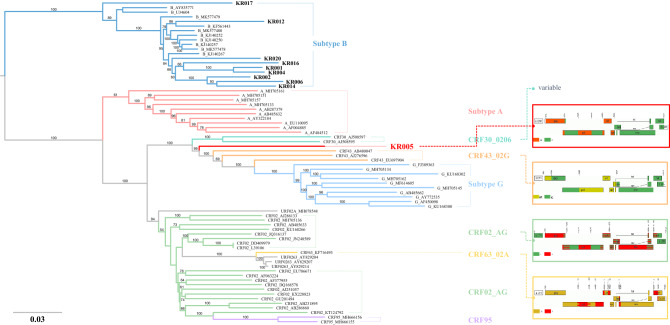
Phylogenetic tree of the recombinant of near full-length genome and genetic mosaic for the recombinant sequences classified using phylogeny. Maximum likelihood tree of NFLG KR005 indicated in red. Schematic representations of the breakpoint patterns in the right panel with related CRF references (CRF43_02G, CRF02_AG, and CRF63_02A).

Phylogenetic analysis was performed to determine the genetic relatedness of 10 NFLG obtained by NGS. KR005 was found to be systematically located between subtype A and subtype G, and the remaining samples were clustered into subtype B, in particular, the reported reference sequences from Korea. KR017 was closely related to AY835771 and U34604 in USA (Fig. 1). ML phylogenetic analysis of KR005, the HIV-1 recombinant, showed that the CRF43_02G recombinant had the closest phylogenetic relationship with KR005 (Fig. 2). These results indicate that the parental origin of KR005 includes the CRF43_02G isolates. ML phylogeny analysis of the coding sequence (CDS) of each gene in NFLG showed that KR005 was closely related to subtype A based on the *pol, vif, vpr*, and *vpu* genes, and to subtype G based on the *rev*, *env*, and *nef* genes. The other samples strongly clustered within subtype B (Supplementary Figure S1).

### Estimation of the time to the most recent common ancestor (tMRCA) of HIV-1 recombinants and subtype B strains

**Figure 3:**
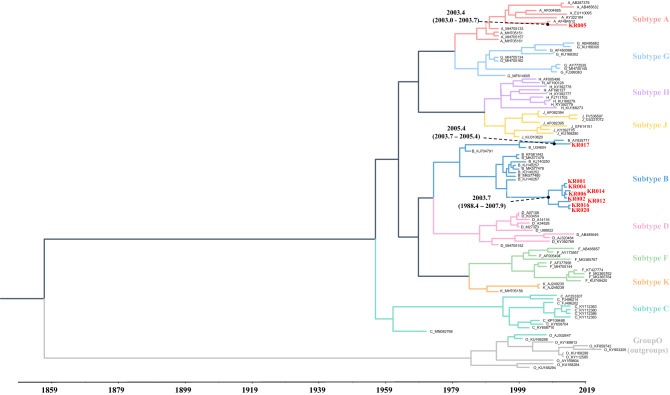
Bayesian analysis of CRF43_02G recombinant sequence and pure B sequences from MSM by NGS. The scale axis below the tree shows the number of years before the present, and the ten clusters identified in our study are indicated by different colors. The ten near full genome sequences are shown in red.

To determine the emergence time of HIV-1 recombinants and subtype B strains using Bayesian molecular clock analysis, ten NFLGs obtained by NGS were analyzed. As shown in Fig. 3, tMRCA of KR005 was estimated to be approximately 16 years before 2019 (divergence time (DT) = 15.6 years; 15.2 to 16.0). The recent origin of the KR005 strains was estimated to be approximately 2003. One subtype B NFLG, KR017, was estimated to have emerged about 14 years ago (DT = 13.6 years; 12.0 to 15.3) from 2019. The other eight subtype B NFLGs originated from the same ancestor approximately 15 years ago (DT = 15.3 years; 11.1 to 20.7) from 2019**.**

### Clinical characteristics by subtype among MSM

We also analyzed the clinical data of twelve participants with NFLG. After 9 years of follow-up (range 0.5‒11 years), two patients with HIV recombinants and ten patients with HIV subtype B had median CD4 + T cell counts of 350 cells/mm^3^ (range 349‒352 cell/mm^3^; follow-up range 2 years) and 582 cells/mm^3^ (range 268‒896 cells/mm^3^; follow-up range 0.5‒11 years), respectively (Supplementary Figure S2). However, no significant difference in CD4 + T cell counts was observed between two groups after follow-up due to small sample size (*p* = 0.2374). The diseases status of all twelve patients during follow-up was investigated. Among the twelve patients who have been observed to multiple diseases, six were with syphilis (50%), five with tuberculosis (42%), three with oropharynx candidiasis disease (25%), and two with condyloma (16%) (Supplementary Table S3). There was still no difference in disease characteristics during follow-up between two groups, the recombinants and subtype B.

## Discussion

Here, we performed, for the first time, the identification of HIV-1 NFLG and the corresponding molecular evolution over time through Bayesian analysis from an HIV-positive MSM population in Korea. Furthermore, for the first time, we identified HIV-1 recombinants using multiple recombination detection methods and phylogenetic analysis. To the best of our knowledge, there is no other report of HIV-1 full genome sequencing from plasma RNA to identify viruses that are currently circulating in Korea. These results show that our methods were appropriate for HIV-1 full genome amplification. Subtype B sequences were the most common form identified using phylogenetic inference based on *env*, *pol*, *gag*, and near-full-length sequences. Monitoring of the HIV-1 genome sequence in the virion is important because virion RNA reflects the currently replicating virus. Analysis of virion RNA is preferable for the study of the pathogenesis and epidemiology of HIV-1 to proviral DNA present in PBMC^10^. Several universal methods for amplifying and sequencing full HIV-1 genomes have been reported^11–13^. However, we adapted the two large amplicons method of Grossmann^12^, in which oligonucleotides have been developed based on simple and efficient protocols for diverse HIV-1 subtypes^10,14^. Furthermore, we first used plasma samples collected from a Korea HIV/AIDS cohort study. Viral RNA was extracted from a large volume (up to 2 mL) and high viral load (> 750,000 copies/mL) of HIV-1 infected plasma or serum specimens to conduct cDNA synthesis and successfully amplify cDNA^10,15^. For viral genome sequencing, the Illumina MiSeq NGS is currently the most widely applied platform, but Illumina DNA polymerases can read ~ 250–300 bp in one direction, with a maximum of 600 bp paired-end reads. In this study, we used the Pacific Biosciences single molecule real-time (SMRT) sequencing technology, which generates extraordinarily long reads of average lengths between 10 and 15 kb^16^. To obtain more full-length sequences, we also used the Sanger sequencing method, although multiple primer pairs were required when analyzing sequences of large segments of HIV-1. In the present study, we were able to obtain a higher proportion of HIV-1 NFLG from samples in our study (24%, 12/50) than in most of the other studies (approximately 10%)^9,17^.

For nearly completed genomes from MSM, the corresponding recombinant events were analyzed using different methods: REGA, jpHMM, RDP4, and ML phylogenetic analysis. Finally, the two near full genome recombinant variants were initially identified in Korea; KR050 as a common CRF07_BC and KR005 as a novel CRF43_02G. However, KR005 showed a close relationship with CRF02_AG in some regions. Thus, this may be an example of a CRF43_02G isolate that recombined with CRF02_AG again, or a new CRF02_AG and subtype G recombinant that came from the same population of viruses where CRF43_02G recombined. CRF43_02G, which was detected in Saudi Arabia in 2008, is an uncommon HIV-1 strain^18^ that was first reported as subtype G in 2007^19^. Therefore, the recombinant from KR005 could be described as a unique recombinant form (URF). However, owing to a lack of epidemiology information, such as where and when KR005 infection first occurred, we could not register it as a new URF or new CRF in the Los Alamos database. Several factors may contribute to the frequency and diversity of recombinant strains, such as co-circulation of multiple HIV-1 subtypes in the same region^20^. CRF07_BC from the KR050 sample was a recombinant form between subtype B-Thai (B') and C and was first described in 2000 ^21^, but was known from western Yunnan, China, in the early 1990s. CRF07_BC is a major circulating recombinant form circulating in China ^22^. For the analysis of clinical data, including CD4 + T cell counts, there was no significant relationship between subtype and disease progression because of the small sample size. A previous study on sequence analysis of *env* gene in HIV-1 Korean B subtype showed that patients with Korean B variants displayed slower disease progression than those with the other subtypes^23^. To understand or identify the viral effects on HIV disease progression, we suggest that a further study with a larger sample size and longer follow-up should be conducted.

In Korea, many epidemiological studies have been conducted using partial genome sequence, such as *gag*, *pol*, or *env* genes, and the proportion of Korean B was reported (87.3%)^7,24,25^. Several studies consistently reported this unique Korean clade B (Korean B), by analyzing various genes such as *nef* ^24^, *env*^26^, *vif* ^27^, and *pol*^28,29^. The Korean B strain has been the predominant strain in Korea for 26 years, since 1985. However, recombination and sequence diversity of a complex genome could not be completely characterized when partial genome sequences were used for HIV-1 subtyping.

In this study, we also performed subtype analysis of *gag*, *pol*, and *env* genes for each clinical sample. Unlike the full-length sequence analysis, the *gag* gene in KR005 was subtyped as A and *env* gene as G. Consequently, full genome sequencing should be performed to confirm the presence of novel circulating recombinants in these samples.

Regarding the Bayesian analysis, the introduction of KR005 sample in 2007 was probably a recent event dated by 2003. We suggested that the CRF43_02G recombinant strains were most likely circulating among Korean MSM since the estimated time by tMRCA.

HIV-1 recombinant viruses are prevalent in areas where multiple subtypes co-circulate, but they are infrequent in Korea where subtype B is predominant. However, to investigate the changes and characteristics of HIV recombinants in Korea, we need to identify and characterize NFLG periodically for cases of HIV diagnosed in Korea. This monitoring of HIV molecular epidemiology would be useful for predicting HIV epidemics and developing strategies for HIV prevention.

In conclusion, we identified a total of twelve HIV-1 NFLGs among fifty MSM plasma samples from a Korea HIV/AIDS cohort study. Two of them were revealed to be a novel CRF43_02G recombinant variant and a common CRF_07BC recombinant, whereas the remainder corresponded to the B subtype. We highlighted the importance of HIV-1 full-length genome analysis for the identification of new recombinant forms and clinical data analysis according to subtype among MSM, known as the major HIV high risk group in Korea. Regarding the recent status of HIV-1 epidemic in Korea, which has increased in terms of cases among young adults, males, and foreigners, there is a need for studies focusing on obtaining full genome sequences to better understand the impact of the viral diversity and dynamics of recombinants. Such events affect most aspects of the HIV pandemic; therefore, further studies are needed to improve the resolution of the HIV-1 genomic diversity and transmission dynamics.

## Methods

### Study samples

Plasma samples and data were obtained from a Korea HIV/AIDS cohort study that was established in 2006 for evidence-based prevention, treatment, and effective management of patients with HIV in Korea^30^. The participants in the cohort were repeatedly surveyed, and blood samples were collected for six or twelve months. Data were managed by the cohort database system and samples were regularly stored in a LN2 tank at − 196 °C at the National Biobank of Korea (NBK) in the Korea Centers for Diseases Control and Prevention (KCDC). For the number of samples of HIV-1-infected MSM from the Korea HIV/AIDS Cohort Study, 50 plasma samples were used based on the following conditions: the male infected due to MSM, the total potential number of samples (> 5 vials), viral load to enhance the sensitivity of amplification (> 10,000 copies/ml). We followed the NBK guidelines to obtain the samples, and this study was approved by KCDC Research Ethics Committee (2017-01-03).

### Viral RNA extraction

Virions were initially concentrated from plasma by Centricon ultrafiltration (Amicon, Burlington, MA, USA). Viral RNA was extracted from 280 μL HIV-1 positive plasma using the QIAmp Viral RNA Mini Kit (Qiagen, Hilden, Germany).

### Reverse transcription

NFLGs of samples were amplified in two fragments using nested PCR with different gene-specific primer sets. Extracted RNA was immediately reverse transcribed to cDNA using the Superscript III first-strand synthesis system (Invitrogen, Carlsbad, CA, USA). Briefly, RNA was primed with 6352R and 9605R (final concentration: 0.6 μM per primer). The reaction mixture was heated to 65 °C for 5 min and maintained at 45 °C until the addition of the second master mix. The final reaction mix was incubated for 2 h at 45 °C, and finally at 85 °C for 5 min to terminate the reaction.

### NFLG PCR and sequencing

NFLG amplification protocol for two regions was performed as described previously, with some modifications^12^. The amplification primers used are listed in Supplementary Table S4. The first fragment (F1), of about 5.5 kb in length, consisted of the *gag* to *vpu* position (776–6231 relative to HXB2); the second fragment (F2), of approximately 3.7 kb in length, included the *vif* to 3LTR position (5861–9555 relative to HXB2). For F1, the first round of PCR was performed with the 682F and 6352R primers, followed by a second round of nested PCR with the 776F and 6231R primers. The F2 fragment was amplified using the 5550F and 9555R primers and 5861F and 9555R primers by semi-nested PCR. Furthermore, the second round primers, which contained a set of 16 nt barcodes, were used for barcoded SMRT sequencing of the near-full-length HIV-1. PCR was performed for 30 cycles at 98 °C for 30 s, 55 °C for 30 s, and 68 °C for 6 min. For F2, the same cycling conditions were used, but with an extension time of 4 min. Two overlapping fragments were amplified using PrimeSTAR GXL DNA polymerase (TaKaRa Bio, Shiga, Japan), and a final primer concentration of 0.4 μM in 50 µl reaction mixtures. PCR products were purified using the direct QIAquick PCR purification kit according to the manufacturer’s instructions (Qiagen). After quality check, the amplicons were sequenced using P6-C4 chemistry on a PacBio RS II instrument (Pacific Biosciences, Menlo Park, CA, USA). Subgenome amplification and sequencing analysis are described in the Supplementary material.

### Data analysis

Based on the HIV-1 reference sequence (HXB2; NCBI accession: K03455^31^), PacBio sequence reads of each sample were mapped using the asm10 algorithm of minimap2 aligner v2.17^32^. To reduce the effects of cross-contamination, reads were filtered. Aligned PacBio sequence reads were assembled to consensus sequences to develop the major genotype using SAMtools v1.9^33^ and freebayes v1.2^34^. Considering the sequencing error rates of PacBio sequencing^35^, genotype variant calling was performed with minimum thresholds of 0.3 for allele frequency, 30 for mapping quality and allele quality, and 100 for mapping coverage. Genetic regions of HIV-1 in the genomic sequences after the consensus assembly were annotated based on the HIV-1 HXB2 reference genome sequence^31^. Sanger sequences from each sample were aligned and assembled using GeneStudio v2.2 (GeneStudio, Inc., Suwanee, GA, USA). To improve the sequence accuracy, low-quality base callings from Sanger sequencing were trimmed out.

### Recombination analysis

HIV-1 consensus sequences were subjected to recombinant analysis to determine their subtype/CRF classification. The mosaic recombinant structure was screened using several different strategies. The REGA HIV-1 subtyping tool v3 (Bioinformatic Bioafrica)^36^ and BPs were verified by the jumping profile hidden Markov model (jpHMM; http://jphmm.gobics.de)^37^. Recombination events were detected using RDP4 package (recombination detection program v.4): RDP^38^, GENECONV^39^, BootScan^40^, MaxChi^41^, Chimera^42^, SiScan^43^, and 3Seq^44^. Only break points detected using more than three methods with *p* < 0.05 were selected. As suggested in previous studies^17,45^, the window size parameters were adjusted to 60 bp in RDP, 120 bp in MaxChi and Chimaera, and 500 bp in BootScan and SiScan; all other parameters were kept at the default values. The partial sequence alignments of each fragment were extracted from the genomic sequence alignment for the following phylogenetic analyses.

### Maximum likelihood (ML) phylogenetic tree inference

Phylogenetic analyses were carried out to classify the isolates by HIV-1 subtype or CRFs. To infer the phylogenetic relationships and positions in HIV-1 group M, phylogenetic analyses on the genome sequences, gene regions, and putative recombinant sequences were performed. Since the output sequences varied in length and quality depending on the methods of sequencing, the sequences obtained with NGS were subjected to phylogenetic analysis. For genomic phylogenetic analysis (Fig. 1), up to ten genomic sequences from each pure subtype of HIV-1 group M, as in group taxa, and ten out-group genomic sequences from HIV-1 group O, were selected. The sequences were aligned with the default set of the ClustalW algorithm in MEGA X v10, followed by manual alignment adjustment. For phylogenetic analyses on gene regions (Supplementary Figure S1), the coding sequences of each gene were translated into their amino acid sequences and aligned with the default set of ClustalW with the BLOSUM matrix in MEGA X^46–48^, followed by manual alignment adjustment. To improve reliability, we selected the results of phylogenetic tree reconstruction with 30 HIV-1 recombinant genome sequences with high similarity sequences, 10 sequences each of subtypes A and G as in-group taxa, and 10 sequences of subtype B for out-group taxa (Fig. 2). The most similar reference sequences were searched by HIV BLAST in the Los Alamos HIV sequence database (Supplementary Table S5). The alignment method used was identical to that used for the genome sequence alignments described above. For ML analyses, IQ-TREE was used with 5000 UFBoot bootstrap replicates^49,50^, and the substitution models for ML analyses in each fragment were selected by ModelFinder in the IQ-TREE pipeline^51^.

### Estimation of the time to the most recent common ancestor for HIV-1 recombinants and subtype B strain divergence

To understand the divergence time and evolutionary time scale of 10 HIV-1 sequences obtained with NGS, molecular clock analysis with Bayesian inference was performed with BEAST v2.6 (burn-in = 25%)^52^. For Bayesian inference, the two most early genomic HIV-1 sequences (MN082768^53^ and KJ704791^54^) were included in the genomic sequence alignment, which was used in the genomic phylogenetic analysis above. Based on a previous study on the origin time and the evolutionary time scale of HIV-1 with partial sequence fragments of *gag*, *pol*, and *env* genes^55^, a relaxed clock lognormal clock model and a coalescent Bayesian skyline tree model were selected with MCMC 10,000,000 generation for the inference. After inferring the monophyletic groups with preliminary analysis without any timescales, subtype-level monophyletic taxa were forced into monophyletic groups to ignore the false close relationships biased by sampling dates. The timescale of HIV-1 genomic evolution was inferred with tip dates of sampling (years); if this was not available, it was replaced by publication years (Supplementary Table S5).

### Statistical analysis

Graphs were generated using Prism 5 software (GraphPad, La Jolla, CA, USA). The Mann–Whitney U test was used to check for statistical significance in our findings.

## Supplementary Information

Supplementary Information.

Supplementary Information.

## Data Availability

NFLG sequences from the study have been deposited in the GenBank with the Accession No. MT021899-MT021910.
