# Supplementary figures and images for: Characterization of HIV-1 recombinant and subtype B near full-length genome among men who have sex with men in South Korea
Source: Sci Rep. 2021 Feb 18;11:4122. doi: 10.1038/s41598-021-82872-3 (PMC7892834; doi:10.1038/s41598-021-82872-3)

Supplementary Figure 1

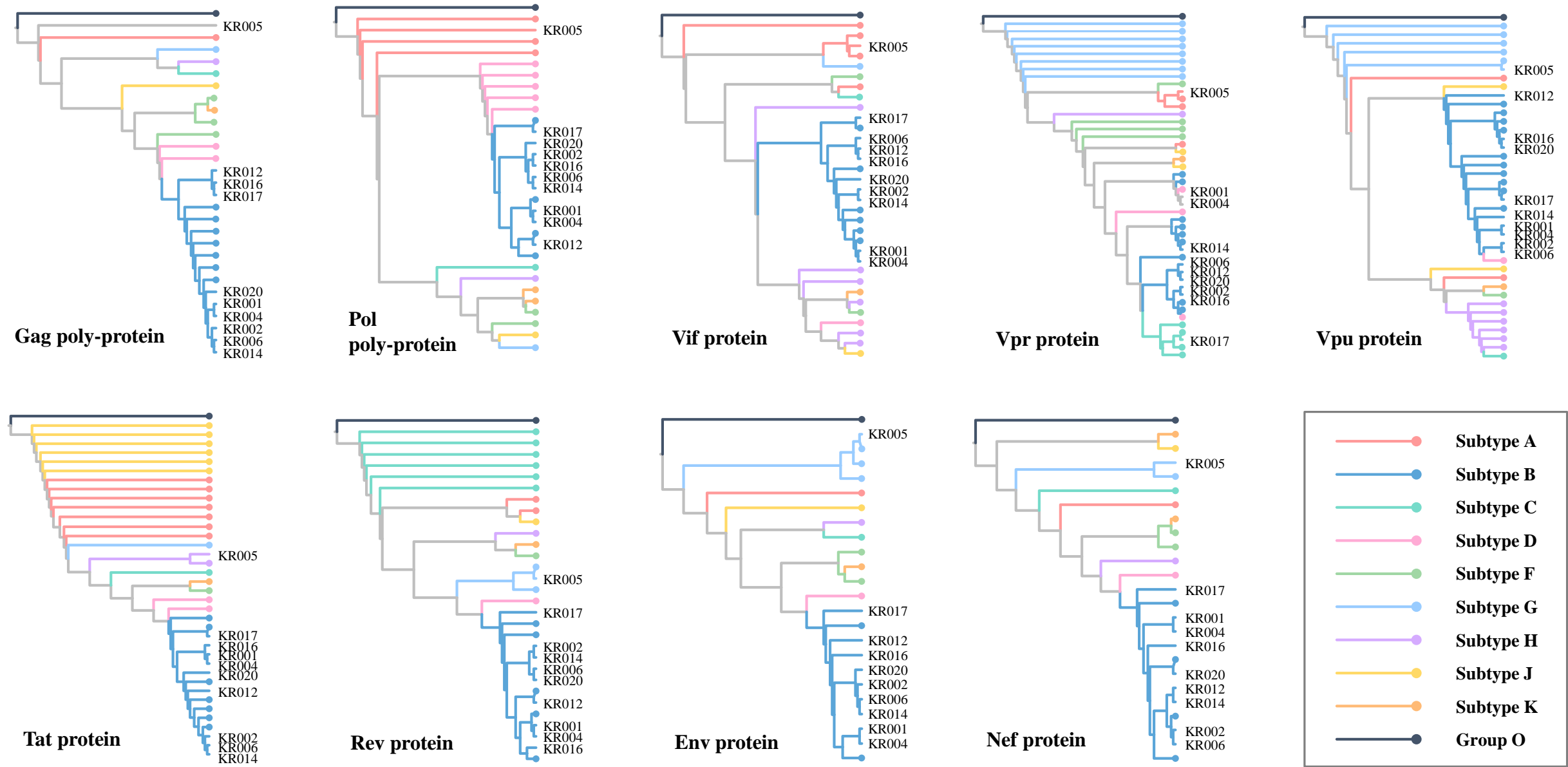

Supplementary Figure 2

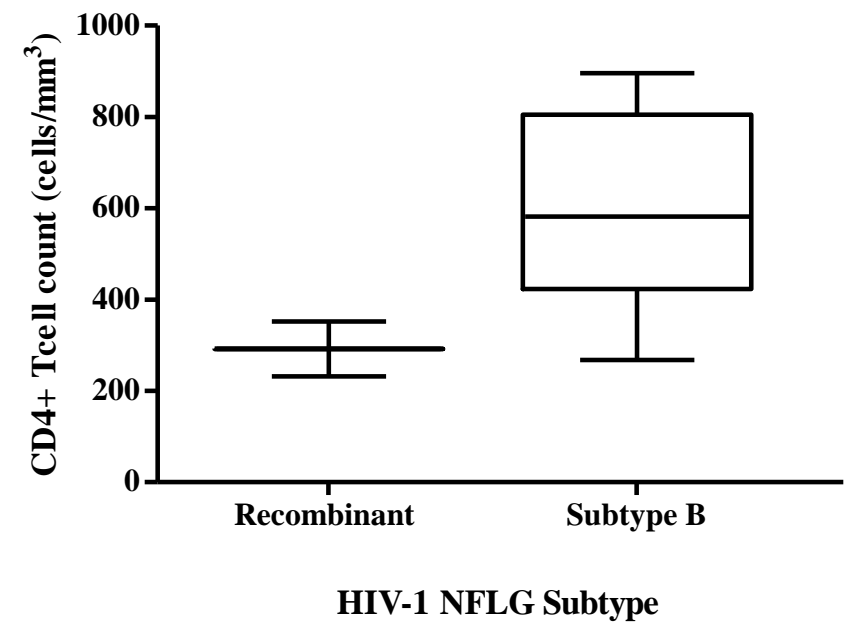

Supplement: Supplementary file 1 — Supplementary Information. [file 41598_2021_82872_MOESM1_ESM.pdf]
