# Supplementary material for: Characterization of HIV-1 recombinant and subtype B near full-length genome among men who have sex with men in South Korea
Source: Sci Rep. 2021 Feb 18;11:4122. doi: 10.1038/s41598-021-82872-3 (PMC7892834; doi:10.1038/s41598-021-82872-3)
Supplement: Supplementary file 2 — Supplementary Information. [file 41598_2021_82872_MOESM2_ESM.docx]

**Characterization of HIV-1 recombinant and subtype B near full-length genome among men who have sex with men in South Korea**

**Sangmi Ryou^1^, Myeongsu Yoo^1^, Kisoon Kim^1^, Sangsoo Kim^2^, Sang Il Kim^3^, Youn Jeong Kim^3^, Dae Won Park^3^, Jun Yong Choi^4^, Hyo Youl Kim^5^, Jung Ho Kim^4^, Joon Young Song^6^, Shin-Woo Kim^7^, Hyun-Ha Chang^7^, Bo Youl Choi^8^, and Mee-Kyung Kee^1^***

^1^ Division of Viral Disease Research, Center for Infectious Diseases Research, Korea National Institute of Health, Cheongju, Korea

^2^ Department of Bioinformatics and Life Science, Soongsil University, Seoul, Korea

^3^ Division of Infectious Disease, Department of Internal Medicine, Seoul St. Mary's Hospital, College of Medicine, The Catholic University of Korea, Seoul, Korea

^4^ Department of Internal Medicine and AIDS Research Institute, Yonsei University College of Medicine, Seoul, Korea

^5^ Department of Internal Medicine, Yonsei University Wonju College of Medicine, Wonju, Korea

^6^ Division of Infectious Disease, Department of Internal Medicine, Korea University College of medicine, Seoul, Korea

^7^ Department of Internal Medicine, School of Medicine, Kyungpook National University

^8^ Department of Preventive Medicine, College of Medicine, Hanyang University, Seoul, Korea

* Corresponding author

E-mail: keemeekyung@gmail.com (M-K)

**Supporting information**

**Supplementary Table S1.** Clinical characteristics data of fifty study participants.

**Supplementary Table S2.** PCR product size and subtype of HIV-1 partial genes.

**Supplementary Table S3.** A clinical follow-up investigations of 12 participants with near full length genome sequences.

**Supplementary Table S4.** Primers used in this study.

**Supplementary Table S5**. HIV-1 genomic reference sequence list for phylogenetic analyses.

**Supplementary Figure S1.** Maximum likelihood phylogenetic tree of separate genomic regions (*gag*, *pol*, *vif*, *vpr*, *tat*, *rev*, *env*) of the ten near full genome sequences.

**Supplementary Figure S2.** Comparison of CD4+ T cell counts by subtype after follow-up for 12 patients.

**Supplementary Table 1.** Clinical characteristics data of fifty study participants.

| **Characteristics** | **Total** | | **12 NFLG** | |
| --- | --- | --- | --- | --- |
|  | **N** | **(%)** | **N** | **(%)** |
| **Age** |  |  |  |  |
| < 20 | 3 | ( 6.0) | 2 | (16.7) |
| 20-39 | 35 | (70.0) | 7 | (58.3) |
| 40-59 | 10 | (20.0) | 3 | (25.0) |
| ≥ 60 | 2 | ( 4.0) | - | - |
| **Viral load^a^** |  |  |  |  |
| < 100,000 | 11 | (22.0) | 1 | ( 8.3) |
| 100,000- 500,000 | 32 | (64.0) | 9 | (75.0) |
| > 500,000 | 7 | (14.0) | 2 | (16.7) |
| **CD4+ cell count (cell/mm^3^)^a^** |  |  |  |  |
| 0-249 | 30 | (60.0) | 9 | (75.0) |
| 250-499 | 16 | (32.0) | 2 | (16.7) |
| 500-1,000 | 3 | ( 6.0) | 1 | 8.3 |
| missing | 1 | ( 2.0) | - | - |
| **Therapy** |  |  |  |  |
| Naïve | 42 | 84.0 | 11 | 91.7 |
| Experience | 8 | 16.0 | 1 | 8.3 |
| **DRM** |  |  |  |  |
| PI | 1 | 2.0 | - | - |
| NRTI/NNRTI | 5 | 10.0 | 1 | 8.3 |
| **AIDS related/defining diseases^b^** |  |  |  |  |
| Yes | 27 | 54.0 | 9 | 75.0 |
| No | 23 | 46.0 | 3 | 25.0 |

NFLG: Near Full Length Genome; DRM: Drug Resistance Mutant; PI: Protease Inhibitor; NRTI: Nucleoside Reverse Transcriptase Inhibitor; NNRTI: Non-nucleoside reverse transcriptase inhibitor. AIDS related/ defining diseases: Tuberculosis; Syphilis; Gonorrhea; Candidiasis; HIV-related encephalopathy; Cytomegalo virus (CMV) disease. ^a^Viral load was measured in copies/ml. CD4+ T was measured in cells/mm^3^. ^b^ diseases diagnosed at entry of a study.

**Supplementary Table S2.** PCR product size and subtype of HIV-1 partial genes.

| ID | *gag* | | *pol* | | *env* | |
| --- | --- | --- | --- | --- | --- | --- |
|  | Product size (bp) | Subtyp^a^ | Product size (bp) | Subtype | Product size (bp) | Subtype |
| KR001 | 505 | B | 1321 | B | 1219 | B |
| KR002 | - | - | 1330 | B | 1139 | B |
| KR003 | - | - | 1042 | B | 1230 | B |
| KR004 | 215 | B | - | - | 1180 | B |
| KR005 | 523 | A(CM) | - | - | 1189 | G(NG) |
| KR006 | 398 | B | 1113 | B | 1121 | B |
| KR007 | 319 | B | 1090 | B | - | - |
| KR008 | 406 | B | 1188 | B | 1227 | B |
| KR009 | 521 | B(US) | 601 | B | 1245 | B |
| KR010 | 495 | B | 1105 | B | 1266 | B |
| KR011 | - | - | - | - | - | - |
| KR012 | 394 | B | 1213 | B | 1262 | B |
| KR013 | - | - | - | - | 1258 | B |
| KR014 | 496 | B | 1109 | B | 1175 | B |
| KR015 | 490 | B | 1085 | B | - | - |
| KR016 | 407 | B(JP) | - | - | 895 | B |
| KR017 | 536 | B(JP) | 768 | B(JP) | 1258 | B(US) |
| KR018 | 407 | B | 1250 | B | - | - |
| KR019 | 394 | B | 1214 | B | - | - |
| KR020 | 547 | B | 1165 | B | - | - |
| KR021 | 532 | B | 1293 | B | - | - |
| KR022 | 544 | B | 1317 | B | 1214 | B |
| KR023 | 534 | B | - | - | 1250 | B |
| KR024 | 544 | B | 1330 | B | 1250 | B |
| KR025 | 546 | B | - | - | 1258 | B |
| KR026 | - | - | - | - | 1124 | B |
| KR027 | 293 | B(CN) | 379 | B(US) | - | - |
| KR028 | 543 | - | - | - | - | - |
| KR029 | 547 | B(JP) | 1325 | B | 1259 | B |
| KR030 | - | - | 952 | B | 1265 | B |
| KR031 | - | - | 1317 | B(JP) | 1026 | B |
| KR032 | - | - | - | - | 1193 | B |
| KR033 | - | - | - | - | - | - |
| KR034 | 575 | B(JP) | 1319 | B | 1225 | B |
| KR035 | 573 | B | 1275 | B | - | - |
| KR036 | 573 | B(US) | 1280 | B(US) | - | - |
| KR037 | 575 | B(US) | 1319 | B | 1268 | B |
| KR038 | 576 | B | 1319 | B | 1273 | B |
| KR039 | 577 | B | 1290 | B | - | - |
| KR040 | 545 | B | 1318 | B | - | - |
| KR041 | 577 | B | 1318 | B | 1274 | B |
| KR042 | - | - | - | - | - | - |
| KR043 | 575 | B | - | - | 1240 | B |
| KR044 | - | - | - | - | - | - |
| KR045 | 565 | AE(TH) | - | - | - | - |
| KR046 | 575 | B | - | - | - | - |
| KR047 | 590 | B | - | - | 1344 | B |
| KR048 | 575 | B | 1327 | B | - | - |
| KR049 | 574 | B | - | - | - | - |
| KR050 | 567 | BC(CN) | 1328 | BC(CN) | - | - |

^a^ country code in parentheses represented the country of the best score sequence; CM, Cameroon, JP, Japan, US, USA, CN, China, TH, Thailand. Reminders have represented Korea.

**Supplementary Table S3.** A clinical follow-up investigations of 12 participants with near full length genome sequences

| ID | Visit  number | Follow up (yr) | Last date | Viral load^a^ | CD4^a^ | CD8^a^ | Overall disease history^b^ |
| --- | --- | --- | --- | --- | --- | --- | --- |
|  |  |  |  |  |  |  |  |
| KR001 | 22 | 11 | 20171120 | 39 | 896 | 926 | - |
| KR002 | 3 | 0.5 | 20070425 | 1,000 | 706 | 133 | Tuberculosis |
| KR004 | 23 | 11 | 20180309 | 56 | 458 | 433 | Hepatitis C, Syphilis, Herpes simplex, Peripheral neuropathy, Oropharynx candidiasis |
| KR005 | 19 | 2 | 20090513 | 27,204 | 352 | 1,159 | Tuberculosis, |
| KR006 | 2 | 0.5 | 20070306 | 115,000 | 268 | 531 | Syphilis |
| KR012 | 22 | 11 | 20180115 | 19 | 776 | 760 | Oropharynx candidiasis |
| KR014 | 21 | 10 | 20170922 | 20 | 478 | 478 | Syphilis |
| KR016 | 21 | 10 | 20180330 | 23 | 569 | 738 | Tuberculosis, Syphilis, Pneumocystosis |
| KR017 | 20 | 10 | 20170703 | 39 | 893 | 687 | Dyslipidemia, CMV, Tuberculosis |
| KR020 | 16 | 8 | 20150119 | 20 | 319 | 1,076 | Oropharynx candidiasis, Syphilis, Tuberculosis, Pneumocystosis |
| KR021 | 14 | 7 | 20170802 | 39 | 594 | 893 | Condyloma accuminatum |
| KR050 | 5 | 2 | 20180410 | ND | 349 | 863 | Condyloma accuminatum, Syphilis |

^a^Viral load was measured in copies/ml. CD4+ T cell count and CD8+ T cell were measured in cells/mm^3^. They were recorded at last visit.

^b^ History of diseases diagnosed during follow up duration; ND, No Data, Observation with multiple responses.

**Supplementary Table S4.** Primers used in this study.

| Set and  Primer | Sequence (5′ -3′ ) | Positions  (nt) | Product size(bp) |
| --- | --- | --- | --- |
| **F1 fragment** |  |  |  |
| First PCR |  |  | 5,670 |
| 682F | TCT CTC GAC GCA GGA CTC GGC TTG CTG | 682-708 |  |
| 6352R | GGT ACC CCA TAA TAG ACT GTR ACC CAC AA | 6352-6323 |  |
| Second PCR |  |  | 5,455 |
| [PB-BC^a^] 776F | CTA GAA GGA GAG AGA GAT GGG TGC GAG | 776-800 |  |
| [PB-BC] 6231R | CTC TCA TTG CCA CTG TCT TCT GCT C | 6231-6207 |  |
| **F2 fragment** |  |  |  |
| First PCR |  |  | 4,005 |
| 5550F | AGA RGA YAG ATG GAA CAA GCC CCA G | 5550-5575 |  |
| 9555R | TCT ACC TAG AGA GAC CCA GTA CA | 9555-9532 |  |
| Second PCR |  |  | 3,694 |
| [PB-BC] 5861F | TGG AAG CAT CCR GGA AGT CAG CCT | 5861-5884 |  |
| [PB-BC] 9555R | TCT ACC TAG AGA GAC CCA GTA CA | 9555-9532 |  |

^a^ PB-BC; PacBio-Barcode, barcode sequences listed in Pacbio system (pacb.com/product-services/). In this study we used barcode ID; 1002F/R, 1004F/R, 1016F/R, 1054F/R, 1055F/R, 1063F/R.

**Supplementary Table S5.** HIV-1 genomic reference sequence list for phylogenetic analyses.

| NCBI accession | Subtype/recombinant type | Sampling date (year) | Publication date (year) | Authors |
| --- | --- | --- | --- | --- |
| AB287379 | subtype A | . | 2006 | Sakamoto,Y. et al. |
| AB485632 | subtype A | . | 2009 | Takekawa,N. et al. |
| AF004885 | subtype A | . | 1998 | Neilson,J.R. et al. |
| AF484512 | subtype A | . | 2002 | Harris,M.E. et al. |
| AY322184 | subtype A | . | 2004 | Fang,G. et al. |
| EU110095 | subtype A | . | 2007 | Land,A.M. et al. |
| MH705133 | subtype A | 1987 | 2018 | Yamaguchi,J. et al. |
| MH705151 | subtype A | 1987 | 2018 | Yamaguchi,J. et al. |
| MH705157 | subtype A | 1987 | 2018 | Yamaguchi,J. et al. |
| MH705161 | subtype A | 1987 | 2018 | Yamaguchi,J. et al. |
| AY835771 | subtype B | . | 2006 | Mikhail,M. et al. |
| KF561443 | subtype B | 1993 | 2013 | Cho,Y.-K et al. |
| KJ140250 | subtype B | 1995 | 2014 | Cho,Y.-K et al. |
| KJ140252 | subtype B | 1992 | 2014 | Cho,Y.-K et al. |
| KJ140257 | subtype B | 1992 | 2014 | Cho,Y.-K et al. |
| KJ140267 | subtype B | 1992 | 2014 | Cho,Y.-K et al. |
| KJ704791 | subtype B | 1979 | 2016 | Worobey,M. et al. |
| MK577478 | subtype B | 1993 | 2019 | Cho,Y.-K et al. |
| MK577479 | subtype B | 1993 | 2019 | Cho,Y.-K et al. |
| MK577480 | subtype B | 1992 | 2019 | Cho,Y.-K et al. |
| U34604 | subtype B | . | 1995 | Guillon,C. et al. |
| AY253307 | subtype C | . | 2004 | Arroyo,M.A. et al. |
| FJ496202 | subtype C | 2003 | 2009 | Salazar-Gonzalez,J.F. et al. |
| FJ496214 | subtype C | 2003 | 2009 | Salazar-Gonzalez,J.F. et al. |
| KP109488 | subtype C | 1999 | 2015 | Hora,B. et al. |
| KY112363 | subtype C | 2008 | 2017 | Iyer,S.S. et al. |
| KY112380 | subtype C | 2008 | 2017 | Iyer,S.S. et al. |
| KY112383 | subtype C | 2008 | 2017 | Iyer,S.S. et al. |
| KY112386 | subtype C | 2008 | 2017 | Iyer,S.S. et al. |
| KY658704 | subtype C | 2000 | 2017 | Hora,B. et al. |
| KY658710 | subtype C | 1996 | 2017 | Hora,B. et al. |
| MN082768 | subtype C | 1966 | 2019 | Gryseels,S. et al. |
| A07108 | subtype D | . | 1993 | Alizon,M. et al. |
| A14116 | subtype D | . | 1996 | Wain-Hobson,S. et al. |
| A34828 | subtype D | . | 1996 | . |
| AB485649 | subtype D | . | 2009 | Takekawa,N. et al. |
| AJ320484 | subtype D | . | 2002 | Novelli,P. et al. |
| K03454 | subtype D | . | 1993 | Alizon,M. et al. |
| KY392769 | subtype D | 2002 | 2017 | Rodgers,M.A. et al. |
| M27323 | subtype D | . | 1993 | Spire,B. et al. |
| MH705152 | subtype D | 1987 | 2018 | Yamaguchi,J. et al. |
| U88822 | subtype D | . | 1997 | Gao,F. et al. |
| AB485657 | subtype F | . | 2009 | Takekawa,N. et al. |
| AF005494 | subtype F | . | 1998 | Gao,F. et al. |
| AF377956 | subtype F | . | 2001 | Carr,J.K. et al. |
| AY173957 | subtype F | . | 2003 | Hierholzer,J. et al. |
| KT427774 | subtype F | 2010 | 2016 | Pessoa,R. et al. |
| KU749420 | subtype F | 2011 | 2016 | Hora,B. et al. |
| MG365762 | subtype F | 2012 | 2018 | Marques,B.C. et al. |
| MG365764 | subtype F | 2012 | 2018 | Marques,B.C. et al. |
| MG365767 | subtype F | 2008 | 2018 | Marques,B.C. et al. |
| MH705144 | subtype F | 2001 | 2018 | Yamaguchi,J. et al. |
| AB485662 | subtype G | . | 2009 | Takekawa,N. et al. |
| AF450098 | subtype G | . | 2001 | Delgado,E. et al. |
| AY772535 | subtype G | . | 2005 | Holzmayer,V. et al. |
| FJ389363 | subtype G | 2004 | 2009 | Yamaguchi,J. et al. |
| KU168300 | subtype G | 2008 | 2016 | Berg,M.G. et al. |
| KU168302 | subtype G | 2003 | 2016 | Berg,M.G. et al. |
| MF614605 | subtype G | 1989 | 2018 | Murzakova,A. et al. |
| MH705134 | subtype G | 1987 | 2018 | Yamaguchi,J. et al. |
| MH705145 | subtype G | 2005 | 2018 | Yamaguchi,J. et al. |
| MH705162 | subtype G | 1987 | 2018 | Yamaguchi,J. et al. |
| AF005496 | subtype H | . | 1998 | Gao,F. et al. |
| AF190127 | subtype H | . | 1999 | Janssens,W. et al. |
| AF190128 | subtype H | . | 1999 | Janssens,W. et al. |
| FJ711703 | subtype H | 2000 | 2009 | Holzmayer,V. et al. |
| KU168273 | subtype H | 2004 | 2016 | Berg,M.G. et al. |
| KU168279 | subtype H | 2002 | 2016 | Berg,M.G. et al. |
| KY392777 | subtype H | 2001 | 2017 | Rodgers,M.A. et al. |
| KY392778 | subtype H | 2001 | 2017 | Rodgers,M.A. et al. |
| KY392779 | subtype H | 2001 | 2017 | Rodgers,M.A. et al. |
| AF082394 | subtype J | . | 1999 | Laukkanen,T. et al. |
| AF082395 | subtype J | . | 1999 | Laukkanen,T. et al. |
| EF614151 | subtype J | . | 2007 | Abecasis,A.B. et al. |
| FV536597 | subtype J | . | 2010 | Millar,D.S et al. |
| GU237072 | subtype J | . | 2010 | Yamaguchi,J. et al. |
| KU168280 | subtype J | 2003 | 2016 | Berg,M.G. et al. |
| KU310620 | subtype J | 1993 | 2016 | Bartolo,I. et al. |
| KY392776 | subtype J | 2002 | 2017 | Rodgers,M.A. et al. |
| AJ249235 | subtype K | . | 1999 | Triques,K. et al. |
| AJ249239 | subtype K | . | 1999 | Triques,K. et al. |
| MH705156 | subtype K | 1987 | 2018 | Yamaguchi,J. et al. |
| AJ302647 | subtype O | . | 2001 | Toure-Kane,C. et al. |
| AY169804 | subtype O | . | 2003 | Yamaguchi,J. et al. |
| AY169813 | subtype O | . | 2003 | Yamaguchi,J. et al. |
| KF859742 | subtype O | . | 2013 | Hora,B. et al. |
| KU168284 | subtype O | 2003 | 2016 | Berg,M.G. et al. |
| KU168288 | subtype O | 1994 | 2016 | Berg,M.G. et al. |
| KU168294 | subtype O | 1996 | 2016 | Berg,M.G. et al. |
| KU168298 | subtype O | 2006 | 2016 | Berg,M.G. et al. |
| KY112585 | subtype O | 2006 | 2017 | Mack,K. et al. |
| KY953205 | subtype O | . | 2017 | Hora,B. et al. |
| AB231895 | CRF02 | . | 2006 | Tatsumi,M. et al. |
| AB286860 | CRF02 | 2003 | 2006 | Sakamoto,Y. et al. |
| AB485633 | CRF02 | . | 2009 | Takekawa,N. et al. |
| AF063224 | CRF02 | . | 1998 | Carr,J.K. et al. |
| AF377955 | CRF02 | . | 2001 | Carr,J.K. et al. |
| AJ251057 | CRF02 | . | 2000 | Toure-Kane,C. et al. |
| AJ286133 | CRF02 | . | 2000 | Montavon,C. et al. |
| DD409979 | CRF02 | . | 2007 | Poznansky,M.C et al. |
| DQ168578 | CRF02 | . | 2005 | Carr,J.K. et al. |
| EU786671 | CRF02 | 2006 | 2008 | Fernandez-Garcia,A. et al. |
| GU201494 | CRF02 | . | 2010 | Carr,J.K. et al. |
| JN248589 | CRF02 | 2009 | 2011 | Charurat,M. et al. |
| JQ316137 | CRF02 | . | 2012 | Cho,Y.K. et al. |
| KT124792 | CRF02 | 2009 | 2016 | Tully,D.C. et al. |
| KU168266 | CRF02 | 2000 | 2016 | Berg,M.G. et al. |
| KX228823 | CRF02 | 2008 | 2016 | Ragupathy,V. et al. |
| L39106 | CRF02 | . | 1996 | Howard,T.M. et al. |
| MH705136 | CRF02 | 2004 | 2018 | Yamaguchi,J. et al. |
| AJ508595 | CRF30 | . | 2003 | Mamadou,S. et al. |
| AJ508597 | CRF30 | . | 2003 | Mamadou,S. et al. |
| AB480047 | CRF43 | 2003 | 2009 | Takekawa,N. et al. |
| AJ276596 | CRF43 | . | 2000 | Janssens,W. et al. |
| EU697904 | CRF43 | . | 2008 | Badreddine,S. et al. |
| KF716493 | CRF63 | 2008 | 2013 | Hora,B. et al. |
| MH078548 | CRF63 | 2014 | 2018 | Hora,B. et al. |
| MH666155 | CRF95 | 2015 | 2019 | Billings,E. et al. |
| MH666156 | CRF95 | 2015 | 2019 | Billings,E. et al. |
| AY829204 | URF263 | . | 2004 | Carr,J.K. et al. |
| AY829207 | URF263 | . | 2004 | Carr,J.K. et al. |
| AY829214 | URF263 | . | 2004 | Carr,J.K. et al. |

**Supplementary Figure S1. Maximum likelihood phylogenetic tree of separate genomic regions (*gag*, *pol*, *vif*, *vpr*, *tat*, *rev*, *env*) of the ten near full genome sequences.** The legend at the right indicates the colour code for subtype representation. References sequences and study sequences are coloured according to subtype. Phylogenetic tree was generated using MEGA.

**Supplementary Figure S2. Comparison of CD4+ T cell counts by subtype after follow-up for 12 patients.** The CD4+ T cell count was compared using recombinant and subtype B near full length genome sequences.

**Methods**

**Subgenome PCR and Sanger sequencing**

Reverse transcription (RT)-PCR and Sanger sequencing were used to genotype a HIV-1 gag (558 bp), pol (1173 bp), and env (1244 bp) genes, which are at nt679-1237, nt2147-3320, and nt6557-7801, respectively based on HIV-1 HXB2 numbering. After purification, PCR fragments were sequenced. The details of the PCR and sequencing procedures have been described previously ^1,2^. If the PCR failed, alternative primers specific for gene were used.

**References**

1 Kim, G. J. *et al.* National survey of prevalent HIV strains: limited genetic variation of Korean HIV-1 clade B within the population of Korean men who have sex with men. *J. Acquir. Immune Defic. Syndr.* **48**, 127-132, doi:10.1097/QAI.0b013e31816b6ae6 (2008).

2 Chung, Y. S. *et al.* Phylogenetic transmission clusters among newly diagnosed antiretroviral drug-naive patients with human immunodeficiency virus-1 in Korea: A study from 1999 to 2012. *PLoS One* **14**, e0217817, doi:10.1371/journal.pone.0217817 (2019).
